# Comparison of aqueous, polyethylene glycol-aqueous and ethanolic propolis extracts: antioxidant and mitochondria modulating properties

**DOI:** 10.1186/s12906-018-2234-5

**Published:** 2018-05-23

**Authors:** Loreta Kubiliene, Aiste Jekabsone, Modestas Zilius, Sonata Trumbeckaite, Daiva Simanaviciute, Rima Gerbutaviciene, Daiva Majiene

**Affiliations:** 1 Department of Drug technology and Social Pharmacy, Lithuanian university of Health Sciences, Sukileliu st. 13, LT-50166 Kaunas, Lithuania; 2 Laboratory of Molecular Neurobiology, Neuroscience Institute, Lithuanian University of Health Sciences, Eiveniu str. 4, LT-50009 Kaunas, Lithuania; 3 Department of Clinical Pharmacy, Lithuanian university of Health Sciences, Sukileliu st. 13, LT-50166 Kaunas, Lithuania; 4 Laboratory of Biochemistry, Neuroscience Institute, Lithuanian University of Health Sciences, Eiveniu str. 4, LT-50009 Kaunas, Lithuania; 5 Department of Pharmacognosy, Lithuanian university of Health Sciences, Sukileliu st. 13, LT-50166 Kaunas, Lithuania; 6 Clinical Department, Lithuanian university of Health Sciences, Eiveniu st. 2, LT-50166 Kaunas, Lithuania

**Keywords:** Water, Ethanol, Polyethylene glycol, Extract of propolis, Polyphenols, Radical scavenging activity, Superoxide, Mitochondrial respiration, C6 glioma cell culture

## Abstract

**Background:**

Propolis is multicomponent substance collected by honeybees from various plants. It is known for numerous biological effects and is commonly used as ethanolic extract because most of active substances of propolis are ethanol-soluble. However, water-based propolis extracts could be applied more safely, as this solvent is more biocompatible. On the other hand, water extracts has significantly smaller range and quantity of active compounds. The extraction power of water could be enhanced by adding co-solvent which increases both solubility and penetration of propolis compounds. However, variation of solvents results in different composition of active substances that might have distinct effects. The majority of biological effects of propolis are attributed to the antioxidant properties of its active compounds. Antioxidant effect might be a result of either direct scavenging of ROS or modulation of ROS producing organelle activity. Therefore, the aim of this study was to investigate and compare chemical composition, antioxidant properties and effects on mitochondrial respiration of aqueous (AqEP), polyethylene glycol-aqueous (Pg-AqEP) and ethanolic (EEP) propolis extracts.

**Methods:**

Chemical composition of propolis extracts was determined using HPLC and Folin-Ciocalteu method. Ability to neutralize H_2_O_2_ and intracellular ROS concentration in C6 glioma cells were determined fluorometrically by using 10-acetyl-3,7-dihydroxyphenoxazine and 2′,7′-dichlorofluorescein diacetate, respectively. Mitochondrial superoxide generation was assessed under fluorescent microscope by using MitoSOX Red. Oxygen uptake rates of mitochondria were recorded by high-resolution respirometer Oxygraph-2 k.

**Results:**

Our data revealed that phenolic acids and aldehydes make up 40–42% of all extracted and identified compounds in AqEP and Pg-AqEP and only 16% in EEP. All preparations revealed similar antioxidant activity in cell culture medium but Pg-AqEP and EEP demonstrated better mitochondrial superoxide and total intracellular ROS decreasing properties. At higher concentrations, AqEP and EEP inhibited mitochondrial respiration, but Pg-AqEP had concentration-dependent mitochondria-uncoupling effect.

**Conclusions:**

Aqueous and non-aqueous propolis extracts differ by composition, but all of them possess antioxidant properties and neutralize H_2_O_2_ in solution at similar efficiency. However, both Pg-AqEP and EEP were more effective in decreasing intracellular and intramitochondrial ROS compared to AqEP. At higher concentrations, these preparations affect mitochondrial functions and change energy production in C6 cells.

## Background

Bees collect exudates from different plant sources and primarily use them to patch holes, seal cracks and build panels in beehives and protect them from bee pathogens [1]. The chemical composition of propolis is very variable because it depends on the local flora and specimens from different geographical and climatic areas. Nowadays, there are detected more than 600 chemical compounds presented in this natural product [2]. Numerous combinations of chemical compounds and concentrations of propolis examples result in a large and diverse biological activity [3]. For these properties, propolis was well-known in folk medicine and effectively used for treatment of various human diseases [4]. Furthermore, the amount of research done on propolis during last decades demonstrates a vast spectrum of its’ biological activity, such as antiseptic, anti-inflammatory, antioxidant, antibacterial, antifungal, antineoplastic, hepatoprotective, cardioprotective and immunomodulatory [5, 6].

The constituents of propolis are flavonoids, phenolic acids, terpenes, aromatic acids and others, majority of which are lipophilic, and therefore are easily dissolved in ethanol and methanol. That is why propolis is commonly used as a liquid ethanolic extract [7]. Ethanolic extracts are convenient for certain purposes of external use [8]. However, strong and unpleasant taste as well as irritating solvent limits peroral administration capacity of this extract, does not allow to use it for ophthalmic and pediatric cases and decreases the applicability in pharmaceutical and cosmetics industry. For wider applicability of propolis, aqueous or aqueous-based complex solvents with 20% polyethylene glycol were produced [9]. Water extracts are the most tissue-friendly, however, in such preparations the levels of biologically active compounds were found to be 10–20 times lower compared to ethanolic extract of propolis (EEP). Thus, adding co-solvents to water solvent and modifying production conditions, higher levels of active compounds are achieved. However, different solvents can extract different biologically active compounds resulting distinct biological effects [7]. For this reason, it is relevant to investigate and compare quantities of active compounds and biological effects of aqueous and non-aqueous propolis extracts.

Most of pharmacological effects of propolis are related to antioxidant properties of this product [10]. There is scientific evidence showing that all types of propolis (European, Brazilian, Turkish) have great levels of antioxidant activity. Chemical analysis experiments (α, α-diphenyl-β-picrylhydrazyl (DPPH) and 2,2′-azino-bis(3-ethylbenzthiazoline-6-sulphonic acid) (ABTS) free radical scavenging method) demonstrate that propolis and it’s extracts have free radical scavenging and metal chelating properties [11, 12]. However, antioxidative action in a living system could be performed not by one particularly determined mechanism but by several synergistic pathways, such as direct neutralization of reactive oxygen species (ROS) together with suppression organelles that intracellularly produce ROS [13]. There is very little experimental evidence showing propolis antioxidant effects in living systems [14, 15], and no investigation has been done on evaluation of different propolis preparations on intracellular and intramitochondrial ROS production.

The aim of this study was (1) to compare the chemical composition of aqueous and non-aqueous propolis extracts, (2) to investigate their direct antioxidant activity in cell culture and intracellular medium and (3) to evaluate the effect of different propolis extracts on mitochondrial superoxide generation and oxygen consumption.

## Methods

### Chemicals

All solvents, reagents, and standards used in this study were of analytical grade. Vanillic, caffeic, ferulic, p-coumaric acids and vanillin, 2′,7′-dichlorodihydrofluorescein diacetate (DCFH_2_-DA), penicillin-streptomycin solution, trypsin-EDTA solution 0.25% were obtained from Sigma-Aldrich Chemie GmbH (Steinheim, Germany). HPLC-grade acetonitrile, trifluoroacetic acid (TFA) and acetic acid (glacial) were obtained from Sigma-Aldrich GmbH (Buchs, Switzerland). Dulbecco’s modified Eagle’s medium (DMEM) with Glutamax, fetal bovine serum were obtained from Gibco (UK); MitoSOX and Mitotracker Green were purchased from Invitrogen (Eugene, USA).

### Propolis raw material and preparation of extracts

Propolis was collected in early autumn, 2016, in Lithuania. The main plants were: *Populus alba*, *P. nigra*, *P. tremula*, *Salix alba*, *S. cinerea*, *Betula verucosa, Pinus silvestris* and *Aesculus hippocastanum*. Voucher specimen (LS 1–16/27089/1R) is deposited at the Department of Drug technology and social pharmacy, Faculty of Pharmacy, Lithuanian University of Health Sciences, Kaunas, Lithuania.

Prior to analysis, propolis samples were kept at room temperature in the dark. Crude propolis was grounded into powder and macerated in different solvents (water, 20% Pg/water and 70% ethanol) by shaking. Extraction time - 5 h in room temperature. Propolis sample-to-solvent ratio was 1:10 (*w*/*v*). After extraction, extracts of propolis were filtered through paper filter and stored at 4 °C. Solutions are clear, yellow liquids and remain stable when stored.

### Determination of total amount of phenolic compounds

The total amount of phenolic compounds in propolis extracts was determined spectrophotometrically using the Folin-Ciocalteu method [16] with slight modifications. Every investigated extract (0.5 ml) was mixed with 2 N 0.5 ml of Folin-Ciocalteu reagent for 6 min. After the addition of 1.5 ml 20% Na_2_CO_3_, the volume was made up to 10 ml with corresponding extraction solvents, followed by incubation for 2 h at room temperature. Absorbance of the mixture was measured at 765 nm using spectrophotometer Agilent 8453 UV-Vis (Agilent Technologies Inc., Santa Clara, USA). Total phenolic compounds content was calculated from the calibration curve of p-coumaric acid and expressed as milligram of p-coumaric acid equivalents per ml of propolis extract.

### Analysis of extracts by high-performance liquid chromatography

Propolis extracts were analyzed using Agilent 1260 Infinity capillary LC (Agilent Technologies, Inc., Santa Clara, CA, USA). Validated HPLC method conditions: C18 column (150 × 0.5 mm, 5 μm particle size); solvent A was acetonitrile and solvent B was 0.5% (*v*/v) of acetic acid in ultrapure water; the linear elution gradient varied from 1 to 21% solvent A for 25 min; the injection volume was 0.2 μl; the flow rate was 20 μl/min; the column temperature was 25 °C. The integration of phenolic compound (vanillic, caffeic, p-coumaric, ferulic acids and vanillin) peaks was performed at 290 nm [17].

### Cell culture

Rat glioma C6 cells were purchased from the Cell Lines Service GmbH (Germany). C6 cells were seeded in culture flasks containing DMEM with 10% of fetal bovine serum, 100 U/ml penicillin and 100 μg/ml streptomycin. The cultures were then incubated at 37 °C, with 5% CO_2_ and saturated humidity; culture transfer was performed once every 3–4 days and cells reseeded to new flask when found confluent.

### Measurement of H_2_O_2_ concentration in cell culture medium

The H_2_O_2_ amount was determined fluorometrically using 10-acetyl-3,7-dihydroxyphenoxazine (Amplex® Red). In combination with horseradish peroxidase, this dye reacts with H_2_O_2_ in a 1:1 stoichiometry to produce the red-fluorescent resorufin. Propolis extracts at concentrations 1, 5, 10 and 15 μg/ml of phenolic compounds (PC) were added to Hank’s balanced salt solution (HBSS) with 50 μM of H_2_O_2_. After treatment with extracts, the remaining part of the H_2_O_2_ reacted with Amplex® Red (5 μM) in the presence of horseradish peroxidase (HRP; 2 U/ml). The fluorescence intensity of resulted resorufin was detected by fluorometer Ascent Fluoroskan (Thermo Fisher Scientific, Inc.) at excitation and emission wavelengths of 544 and 590 nm, respectively. For control, the level of H_2_O_2_ was determined in HBSS containing appropriate amounts of the solvents only.

### Measurement of intracellular ROS concentration

The production of ROS was assessed using the 2′,7′-dichlorofluorescein diacetate (DCFH-DA). After incubation of C6 cells in 96-well plates (20 000 cells/well) for 24 h, they were incubated with DCFH-DA (10 μM) in HBSS at 37 °C for 30 min. During this time a part of DCFH is diffused into the cells. The excess dye was washed out twice with phosphate buffered saline (PBS). Wells were filled with a HBSS medium and extracts at different concentrations (1–15 μg/ml PC) were added. In the presence of cellular oxidizing agents, DCFH is oxidized to the highly fluorescent compound dichlorofluorescein (DCF), thus, fluorescence intensity is proportional to the amount of ROS produced in the cells. The fluorescence of DCF was detected by fluorometer at excitation and emission wavelengths of 488 and 525 nm, respectively. For controls, the level of intracellular ROS was determined using appropriate amounts of different solvents only.

### Detection of mitochondrial superoxide production

The generation of mitochondrial superoxide was assessed using MitoSOX Red. This dye is used for selective detection of superoxide in the mitochondria of living cells. After incubation of C6 cells in 24-well plates (50000 cells/well) for 24 h, they were incubated with MitoTracker Green FM (60 nM, 45 min) for identification of mitochondria and with MitoSox Red (5 μM, 15 min) for superoxide. After incubation, cells were washed twice with PBS. Wells were filled with a HBSS medium with extracts at different concentrations (5–15 μg/ml PC) and incubated for 15 min. Solvent only-treated cells served as controls and Antimycin A (20 μM) treatment was used as positive control. After incubation, cells were washed with HBSS and images were acquired under fluorescence microscope OLYMPUS IX71S1F-3 (Olympus Corporation, Tokyo, Japan). Images were analyzed by ImageJ freeware and data presented as average fluorescence intensity per cell and shown as averages ± SE of 3–5 experiments.

### Assessment of mitochondrial oxygen consumption

The oxygen uptake rates of mitochondria in C6 cells were recorded using a high-resolution respirometer Oxygraph-2 k (Oroboros Instruments, Innsbruck, Austria). Measurement medium contains 0.5 mM EGTA, 3 mM MgCl_2_, 60 mM K-lactobionate, 20 mM Taurine, 10 mM KH_2_PO_4_, 20 mM HEPES, 110 mM sucrose (pH 7.1 at 37 °C). In the beginning of experiment, C6 cells were added into the respirometric chamber and permeabilized with digitonin (10 μg/ml). After cell permeabilization, non-phosphorylating respiration rate was measured with mitochondrial I complex substrates - glutamate (5 mM) and malate (5 mM) or complex I + II substrates - glutamate (5 mM), malate (5 mM) and succinate (12 mM). For investigation of the effect of different extracts on non-phosphorylating respiration rate, the extracts at concentration range 10–120 μg/ml PC were added into the chamber and respiration rate was recorded. Mitochondrial respiration rates were expressed as pmol O_2_/(s*1 × 10^6^ cells/ml).

Control experiments were carried out by registering mitochondrial respiration parameters using solvents only. Solvent quantities were equal to the quantities of added extracts. Results showed no changes in mitochondrial respiration rates after addition of solvents alone.

### Statistical analysis

Results are presented as means ± standard error of 3–5 experiments. Statistical analysis was performed by one-way analysis of variance (ANOVA), followed by Dunnett’s post-test using the software package SigmaPlot 12.0 version (Systat Software Inc.). A value of *p* < 0.05 was taken as the level of significance.

## Results

### Chemical composition of aqueous, polyethylene glycol-aqueous and ethanolic propolis extracts

Results of chemical composition of investigated extracts obtained by HPLC method and total phenolic compound content obtained by Folin-Ciocalteu method are shown in Table 1. Results revealed that the largest amounts of phenolic compounds were determined in EEP; they were 10 and 17 times higher compared to Pg-AqEP and AqEP, respectively. Meanwhile, total amount of phenolic acids and aldehyde vanillin was 4 and 7 times higher compared to Pg-AqEP and AqEP, respectively. Vanillin and p-coumaric acid were dominating compounds in all the extracts investigated.

**Table 1:** Quantity of identified active compounds in aqueous, polyethylene glycol-aqueous and ethanolic propolis extracts

Active compounds ± SE (μg/mL)						Total amount of phenolic acids and vanillin (μg/ml)	Total content of phenolic compounds, (μg/ml)
Extract type	Vanillic acid	Caffeic acid	Vanillin	p-Coumaric acid	Ferulic acid		
AqEP	109.5 ± 10.7	7.5 ± 0.1	156.6 ± 21.4	165.2 ± 10.3	78.4 ± 2.7	514.7 ± 40.6	1 207.9 ± 27.6
Pg-AqEP	151.3 ± 10.4^*^	12.7 ± 2.8^*^	259.7 ± 6.0^*^	298.3 ± 20.2^*^	161.9 ± 5.1^*^	879.6 ± 29.8^*^	2 149.5 ± 16.1^*^
EEP	501.3 ± 23.3^*^	48.1 ± 10.2^*^	888.9 ± 43.0^*^	1167.4 ± 6.2^*^	798.9 ± 54.3^*^	3404.7 ± 124.8^*^	20 791.3 ± 2320.9^*^

^*^P < 0.05, vs AqEP

### H_2_O_2_ neutralising capacity of propolis extracts

**Fig. 1:**
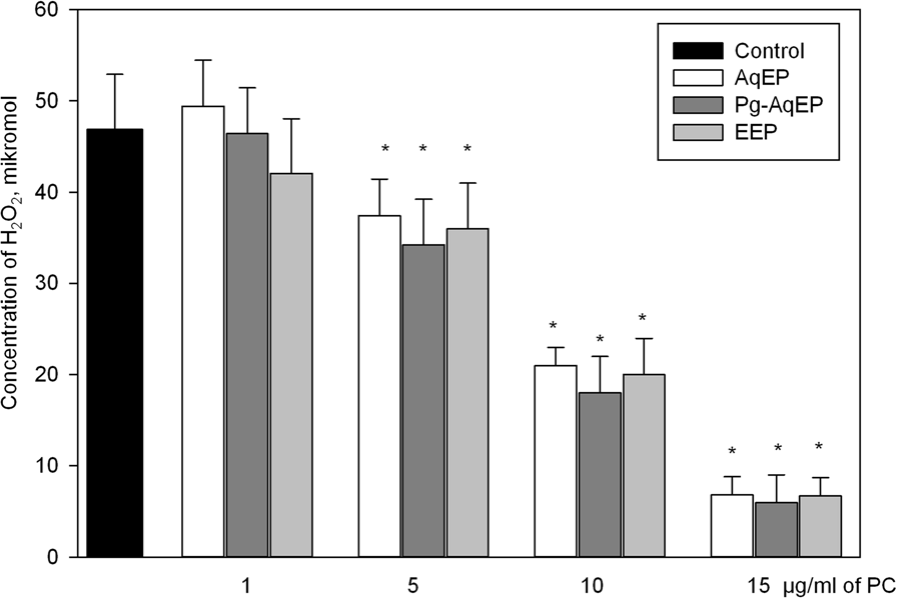
Effect of propolis extracts on H_2_O_2_ concentration in cell culture medium. Different concentrations (1–15 μg/ml PC) of AqEP, Pg-AqEP and EEP were added to wells filled with HBSS and enriched with 50 μM H_2_O_2_. Immediately before measurement horseradish peroxidase and Amplex Red dye were added and fluorescence intensity were measured. Data are presented as means of percentage of the control cells ± SE (*n* = 5). * *p* < 0.05 versus control

The aim of this experiment was to evaluate H_2_O_2_ neutralizing capability of investigated extracts. Results in Fig. 1 show that phenolic compounds extracted using different solvents decreased ROS concentration similarly when applied at equal quantities: 1 μg/ml PC did not significantly reduced H_2_O_2_; 5 μg/ml PC decreased H_2_O_2_ amount by 20–29%; 10 μg/ml PC by 55–62%; and 15 μg/ml PC by 85–87%.

### The effect of propolis extracts on intracellular ROS concentration in C6 cell culture

**Fig. 2:**
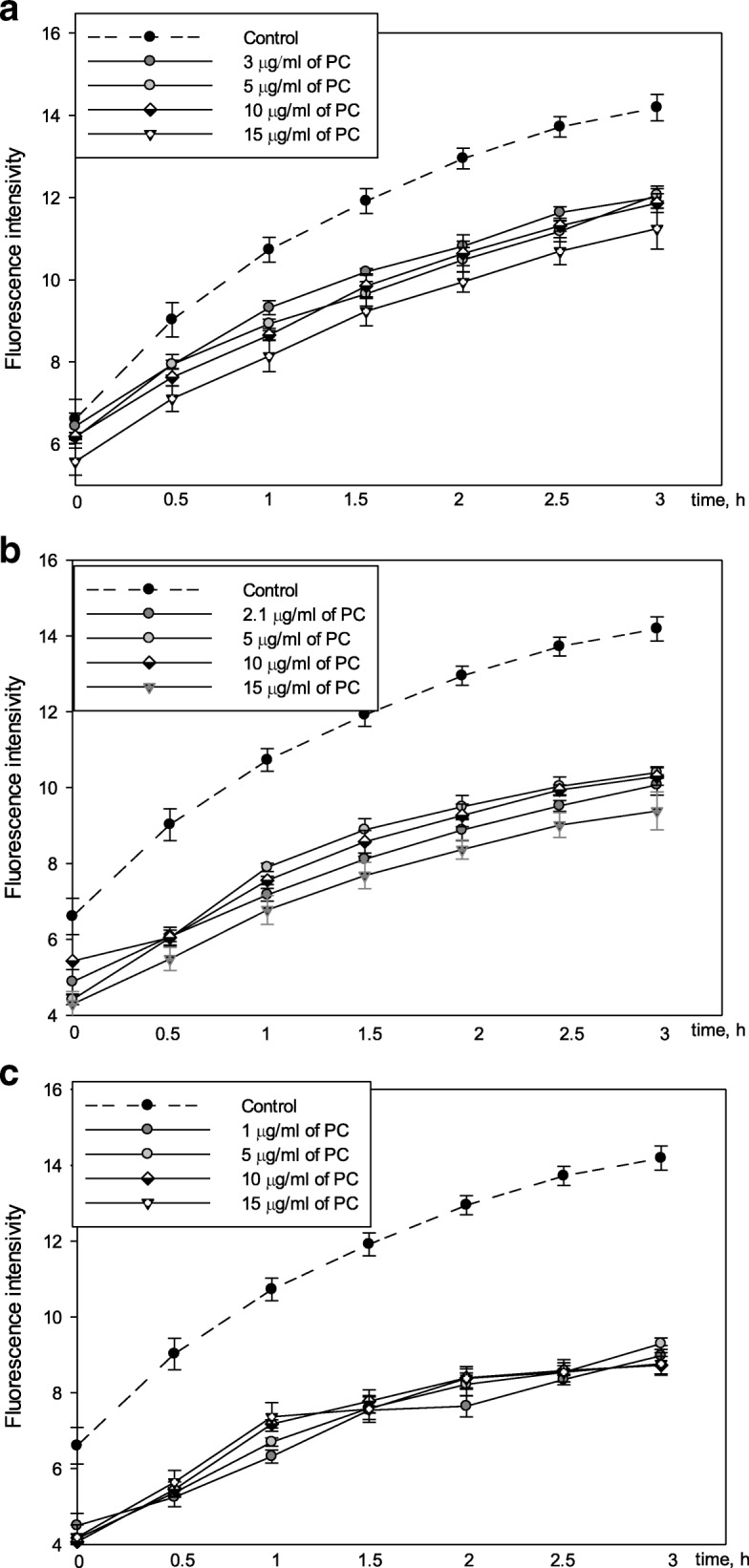
Effect of propolis extracts on intracellular ROS concentration **a** – AqEP, **b** *-* Pg-AqEP, **c** – EEP. C6 cells were pre-treated with 10 μM DCFH-DA and then were treated with different concentrations (1–15 μg/ml PC) of AqEP, Pg-AqEP and EEP. Fluorescence intensity, which is proportional to intracellular ROS concentration, was detected by a fluorometer at excitation and emission wavelengths of 544 and 590 nm, respectively. Data are presented as means of percentage of control cells ± SE (n = 5)

The aim of these experiments was to evaluate how intracellular ROS would be affected by different propolis extracts. Results in Fig. 2a show that AqEP at different concentrations ranging from 3 to 15 μg/ml PC had similar ROS scavenging effect starting from 9 to 10% of total ROS after 0.5 h and ending with 14–18% after 3 h of monitoring. 2.1–15 μg/ml PC Pg-AqEP showed stronger antioxidant activity compared to AqEP reaching 30–35% of total ROS scavenged after 3 h (Fig. 2b). The strongest antioxidant activity was observed with EEP: 15 μg/ml PC quenched 38–43% of intracellular ROS during the same time of monitoring (Fig. 2c).

### Effect of extracts on mitochondrial superoxide concentration in C6 cells

**Fig. 3:**
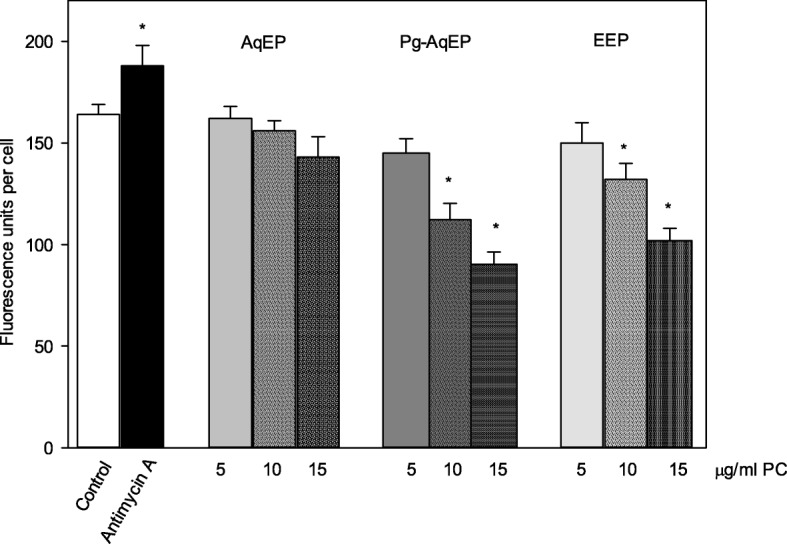
Effect of propolis extracts on mitochondrial superoxide generation in C6 cells. C6 cells were pre-treated with MitoTracker Green FM (60 nM) for 45 min and with MitoSox Red (5 μM) for 15 min. After incubation, cells were treated with different concentrations (1–15 μg/ml) of AqEP, Pg-AqEP and EEP and incubated for 15 min. Solvent only-treated cells served as controls. Antimycin A (20 μM) treated cells were used as positive control. Images were acquired under fluorescence microscope and analyzed by ImageJ freeware. Data are presented as means of percentage of control cells ± SE . * *p* < 0.05 versus control

**Fig. 4:**
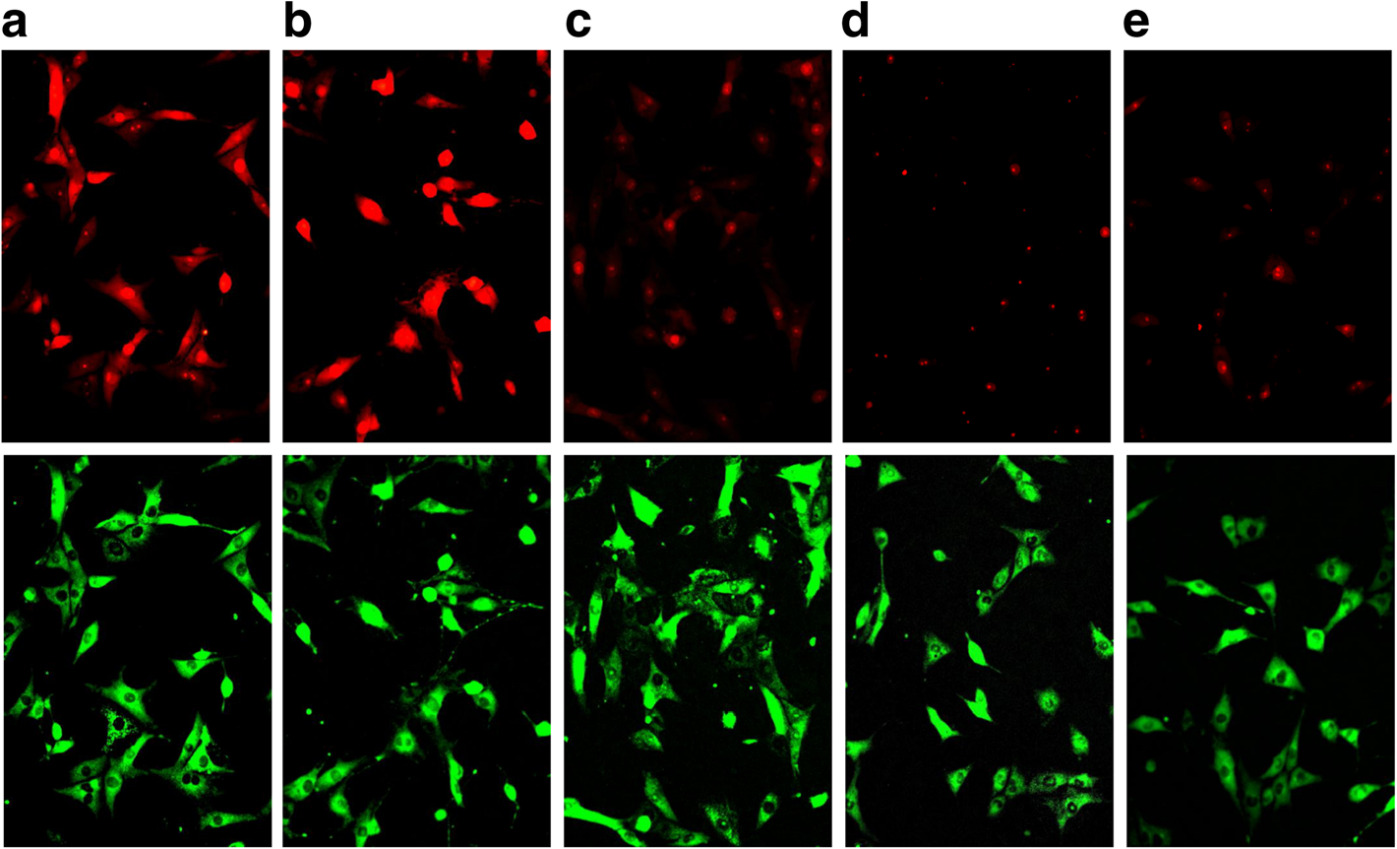
Typical images demonstrating effect of propolis extracts on mitochondrial superoxide generation in C6 cells: **a** - control; **b** - 20 μM of Antimycin A; **c** - 15 μg/ml PC of AqEP; **d** - 15 μg/ml PC of Pg-AqEP; **e** - 15 μg/ml PC of EEP

A mitochondrial respiratory chain complex III inhibitor Antimycin A (20 μM) was used to initiate superoxide generation in mitochondria and increased superoxide level by 15% compared to control (Figs. 3 and 4). In all the samples with added extracts (5–15 μg/ml PC) intramitochondrial superoxide was found decreased, but the extend of the effect was different. The largest superoxide decreasing effect was shown by Pg-AqEP; fluorescence intensity decreased by 12–45% after 15 min incubation with 5–15 μg/ml PC. Slightly smaller effect (by 9–37%) was observed with EEP, and the weakest - with AqEP (1–13%).

### The effect of propolis extracts on mitochondrial non-phosphorylating respiration rate in C6 cells

**Fig. 5:**
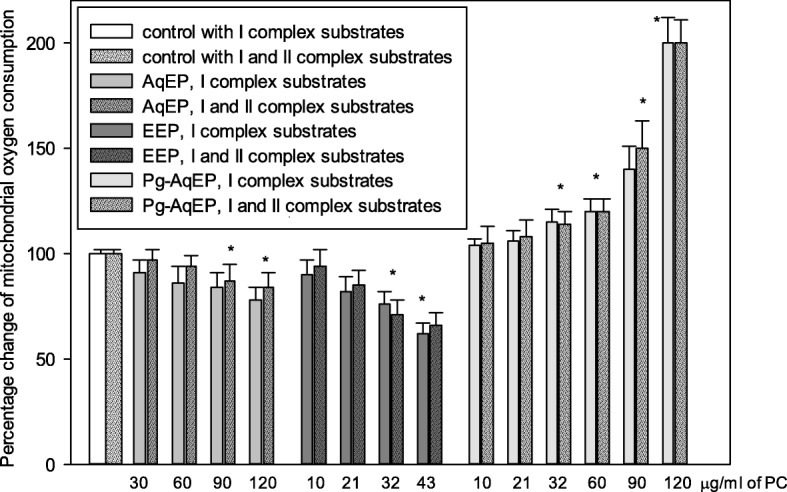
Effect of propolis extracts on mitochondrial non-phosphorylating respiration rate in C6 cells. Incubation of cells and measurement of the digitonin-permeabilised cell mitochondrial respiration were performed as described in Materials and Methods. The respiration rates are expressed as pmol O_2_/(s*2 × 10^6^ cells). Data are presented as means of percentage of control cells ± SE (n = 5). * *p* < 0.05 versus control

The investigation of mitochondrial respiration revealed (Fig. 5) that AqEP used at concentrations up to 30 μg/ml of PC has no effect on mitochondrial non-phosphorylating respiration rate in C6 cells neither with mitochondrial complex I (glutamate and malate), nor complex I and II substrates (glutamate, malate, succinate). AqEP at concentrations 30–60 μg/ml of PC had tendency to reduce non-phosphorylating respiration rate of mitochondria with complex I substrate. 90–120 μg/ml AqEP statistically significantly reduced non-phosphorylating respiration rate by 16–22%. A similar effect has been obtained with mitochondria respiring with I and II mitochondrial complex substrates - 90-120 μg/ml of PC statistically significantly reduced non-phosphorylating respiration rate by 13–16%. EEP had stronger effect on mitochondrial functions compared to AqEP. At lower concentrations (10–21 μg/ml of PC), it had tendency to reduce non-phosphorylating respiration rate both with complex I and I + II substrates. 32–43 μg/ml EEP statistically significantly decreased mitochondrial non-phosphorylating respiration rate by 24–38% and by 29–34% with complex I substrates and complex I + II substrates, respectively. In contrast to AqEP and EEP, Pg-AqEP at 10–21 μg/ml of PC had tendency to increase non-phosphorylating respiration rate both with complex I and I + II substrates. Ar higher concentrations (32–60 μg/ml of PC), Pg-AqEP increased non-phosphorylating respiration rate of mitochondria respiring both with complex I and I + II substrates by 10–25%. At highest investigated concentration (120 μg/ml of PC), Pg-AqEP stimulated the respiration rate be 100%.

## Discussion

Propolis is a resinous substance collected by honeybees from various plants. The chemical composition of propolis is highly variable, depending on the plant sources available to the bees at different locations [16]**.** European propolis is mostly collected from poplar trees and is rich in flavonoids [18]. Biological effects of flavonoids, as the major constituents of propolis as well as ethanolic extract of propolis, have been extensively tested for few decades, therefore our investigations were more focused on the effects of phenolic acids. The results of HPLC experiments showed, that EPP which was produced for our experiment had similar phenolic acid concentration as published by other authors [6, 17]. Phenolic acids and aldehyde vanillin made 42% of all extracted and identified compounds in AqEP and 40% in Pg-AqEP. Moreover, it is important to emphasize that Pg-AqEP has twice more polyphenolic compounds than AqEP. The majority of phenolic compounds (about 80%) presented in EEP were not phenolic acids. Such chemical analysis data about EEP were also demonstrated in studies of other authors. It has been discovered, that the larger part of EEP extracts were made of flavonoids: quercetin, kaempherol, pinocembrin, naringenin, rutin [19, 20].

Antioxidative activity of propolis was repeatedly proven by many researches, however most of experiments were carried out using chemical analysis methods in non-biologically-relevant environment [20]. Our experiments added a new knowledge about antioxidant properties of this substance by revealing propolis ability to reduce ROS in cells and in cell culture medium. All extracts used in our study (Fig. 1) had similar ability to reduce H_2_O_2_ concentration in the medium, and the capacity of this activity was dependent on the concentration of phenolic compounds. Literature data comparing antioxidant properties of AqEP and EEP are controversial. Danert with co-workers demonstrated that propolis samples from Argentina extracted with water presented better radical scavenging ability than ethanolic extracts, independent of the antioxidant method (scavenging activity of ABTS*+, DPPH*, HO* and O2(−)* and β-carotene bleaching test) [21]. However, Park et al. measured antioxidant activity by coupled oxidation of β-carotene and linoleic acid and found that all types of AqEP and EEP possess antioxidant activity, but 70 and 80% EEP demonstrated the greatest effect and AqEP and 20% EEP showed the lowest antioxidant ability [19]. In contrast to the findings described, our study demonstrates that all types of extracts neutralize H_2_O_2_ in similar efficiency, depending on the concentration of phenolic compounds. There is a common tendency to attribute the ability of ROS neutralization to flavonoids, however, our study reveals that phenolic acids have similar or the same ROS-scavenging capacity.

Intracellular ROS are the most harmful oxidants, therefore, it was important to investigate the ability of the extracts to reduce not only extracellular, but also intracellular ROS. While using extracts at equal concentrations of phenolic compounds, AqEP showed the weakest ability to neutralize intracellular oxidants (up to 18% after 3 h treatment), but the effect of Pg-AqEP was twice as strong (up to 35%). It is known that polyethylene glycol might affect cellular membranes by increasing their permeability for chemical compounds [22], therefore it was likely to contribute by easing penetration of antioxidative compounds into the cells and thus increasing its biological efficiency. EEP showed the greatest effect decreasing intracellular ROS concentration (up to 43% after 3 h). Ethanol extracts contain lipophilic substances that can pass through cell membranes easier compared to the hydrophilic ones from aqueous extracts. Results from our experiments with EEP are comparable to studies of other authors. Kamiya with co-workers showed that CdCl_2_ induces cytotoxicity via intracellular ROS accumulation in COS7 cells. Pretreatment with EEP for 1 h significantly reduced intracellular ROS accumulation and suppressed CdCl_2_-induced cytotoxicity [23]. Da Silveira et al. showed that ethanolic extract of yellow Brazilian propolis used at concentrations 1, 3, 10, and 30 mg/kg as dietary supplement for Wistar rats reduced the production of nitric oxide and malondialdehyde without changing level of total antioxidants, catalase, and superoxide dismutase, induced by behavioral stress [14]. It is important to note, that naturally-derived phenolic compounds were often found to switch from antioxidant to prooxidant activity at higher concentrations [24]. In our experiments, however, propolis extracts at concentrations from 1 to 15 μg/ml PC revealed only antioxidant properties. This was also confirmed by other authors, investigating similar (5–50 μg/ml) or even higher concentrations of propolis extracts [25].

Mitochondria use oxygen to make ATP under normal conditions. If cells are exposed to harmful external factors or biologically active substances, mitochondria are one of the first targets to be damaged and consequently might start to reduce molecular oxygen incompletely generating superoxide radicals and become main ROS producers in the cell [26]. Evaluation of propolis extracts as intramitochondrial ROS scavengers revealed that all extracts in concentration dependent manner decreased amount of intramitochondrial superoxide. The strongest effect was observed in the samples incubated with 15 μg/ml PC of EEP and Pg-AqEP (up to 37 and 45%, respectively), but AqEP neutralized only up to 13% of superoxide generated. To our knowledge, there are no literature evidence about the effect of AqEP or Pg-AqEP and only a few reports about the effects of EEP and separate compounds of this extract on intramitochondrial superoxide concentration. Ueda with co-workers demonstrated that Kaempferide and kaempferol, the active ingredients of investigated propolis extract, significantly suppressed mutant SOD1-induced superoxide in mitochondria, and ethanolic extract of brazilian green propolis protected N2a cells against mutant SOD1-induced neurotoxicity. [27]. Benguedouar et al. investigated female Wistar rat liver and heart tissues altered by doxorubicin and vinblastine which stimulate overproduction of mitochondrial superoxide anion. Pretreatment of rats with propolis extract (100 mg/kg/day) administered 4 days prior to doxorubicin (20 mg/kg) and/or vinblastin (2 mg/kg) injection, substantially reduced oxidative stress and protected myocardium and hepatic tissues from the peroxidative damage [28].

Amount of mitochondrial superoxide might decrease not only by direct neutralization, but also by modulating mitochondrial functions. Mitochondrial respiration measurements revealed that AqEP has no influence on mitochondrial function up to 30 μg/ml of PC, therefore it is likely that it has decreased mitochondrial superoxide level due to direct neutralisation of the compound. EEP used at concentrations 10–21 μg/ml of PC had tendency to reduce non-phosphorylating respiration rate both with complex I and I + II substrates (the effect becomes significant at 32 μg/ml of PC). Thus, the extract might affect mitochondrial superoxide level not only due to direct neutralization but also because of decreased oxygen consumption. Pg-AqEP on its turn, had tendency to increase non-phosphorylating respiration rate most likely due to mild uncoupling. Mild mitochondrial uncoupling could be beneficial for cells by protecting intracellular molecules from oxidative stress associated with pathological situations [29]. There is some evidence showing that some flavonoids, phenolic acids and other biologically active compounds (ursolic acid, quercetin, rutin, hyperoside, quercitrin) can partially uncouple mitochondrial oxidative phosphorylation thus reducing mitochondrial ROS generation [30, 31]. Higher concentrations of AqEP and EEP significantly decreased mitochondrial oxygen consumption rate and cellular energy production. Changes in mitochondrial functions may have impact not only on cellular redox status, but also on cellular viability because mitochondria are involved in initiation of apoptosis/necrosis pathways in mammalian cells [32]. It was shown that propolis extracts has cytotoxic activity [2] therefore further studies are needed for investigation of differently prepared propolis extracts on cell viability and clarification of mitochondrial impact on cell death mechanisms.

## Conclusions

Aq and 20% Pg-Aq solvents are able to extract mainly hydrophilic propolis compounds, 40% of which are made of phenolic acids and aldehydes. Pg-Aq solvent extracts 2 times more of phenolic compounds compared to Aq preparation. EEP contains only 16% of phenolic acids and has 10 times larger content of total phenolic compounds.

All propolis extracts neutralize hydrogen peroxide in a similar phenolic compound amount-dependent manner. Pg-AqEP and EEP have 3 times stronger mitochondrial superoxide and total cell ROS reducing effect than AqEP, most likely because of their direct action on mitochondrial functions next to the direct ROS neutralization. At higher concentrations AqEP and EEP inhibit mitochondrial non-phosphorylating respiration with complex I and I + II substrates glutamate/malate and succinate, however Pg-AqEP increases the respiration rate most probably due to the uncoupling of oxidative phosphorylation.

Of all extracts investigated, AqEP had the smallest amount of active substances and weakest biological effects. Addition of co-solvent Pg increases the solubility of propolis compounds and improves penetration of active substances into cells yet does not affect biocompatibility of the product. Thus, such kind of extract could be recommended not only for biological research but also for design of pharmaceutical products in the cases when EEP applicability is limited.

## Abbreviations

Aq: Aqua/water
AqEP: Aqueous extract of propolis
EEP: Ethanolic extract of propolis
PC: Phenolic compounds
Pg: Polyethylene glycol
Pg-Aq: Aqueous solvent with 20% polyethylene glycol
Pg-AqEP: Aqueous and 20% polyethylene glycol extract of propolis
